# CD49f^high^ Cells Retain Sphere-Forming and Tumor-Initiating Activities in Human Gastric Tumors

**DOI:** 10.1371/journal.pone.0072438

**Published:** 2013-08-28

**Authors:** Hiroshi Fukamachi, Hyang Sook Seol, Shu Shimada, Chikako Funasaka, Kanako Baba, Ji Hun Kim, Young Soo Park, Mi Jeung Kim, Keiji Kato, Mikito Inokuchi, Hiroshi Kawachi, Jeong Hwan Yook, Yoshinobu Eishi, Kazuyuki Kojima, Woo Ho Kim, Se Jin Jang, Yasuhito Yuasa

**Affiliations:** 1 Department of Molecular Oncology, Graduate School of Medical and Dental Sciences, Tokyo Medical and Dental University, Tokyo, Japan; 2 Department of Pathology, Asan Medical Center, University of Ulsan College of Medicine, Seoul, Korea; 3 Department of Surgical Oncology, Graduate School of Medical and Dental Sciences, Tokyo Medical and Dental University, Tokyo, Japan; 4 Department of Human Pathology, Graduate School of Medical and Dental Sciences, Tokyo Medical and Dental University, Tokyo, Japan; 5 Department of Surgery, Asan Medical Center, University of Ulsan College of Medicine, Seoul, Korea; 6 Department of Pathology, Seoul National University College of Medicine, Seoul, Korea; Peking University Health Science Center, China

## Abstract

Identification of gastric tumor-initiating cells (TICs) is essential to explore new therapies for gastric cancer patients. There are reports that gastric TICs can be identified using the cell surface marker CD44 and that they form floating spheres in culture, but we could not obtain consistent results with our patient-derived tumor xenograft (PDTX) cells. We thus searched for another marker for gastric TICs, and found that CD49f^high^ cells from newly-dissected gastric cancers formed tumors with histological features of parental ones while CD49f^low^ cells did not when subcutaneously injected into immunodeficient mice. These results indicate that CD49f, a subunit of laminin receptors, is a promising marker for human gastric TICs. We established a primary culture system for PDTX cells where only CD49f^high^ cells could grow on extracellular matrix (ECM) to form ECM-attaching spheres. When injected into immunodeficient mice, these CD49f^high^ sphere cells formed tumors with histological features of parental ones, indicating that only TICs could grow in the culture system. Using this system, we found that some sphere-forming TICs were more resistant than gastric tumor cell lines to chemotherapeutic agents, including doxorubicin, 5-fluorouracil and doxifluridine. There was a patient-dependent difference in the tumorigenicity of sphere-forming TICs and their response to anti-tumor drugs. These results suggest that ECM plays an essential role for the growth of TICs, and that this culture system will be useful to find new drugs targeting gastric TICs.

## Introduction

Gastric adenocarcinomas are the second leading cause of cancer-related mortality in the world [1]. Although early diagnosis by endoscopic screening and surgical treatment give best therapeutic opportunity for gastric cancer patients, 20 to 40% of the tumor have been diagnosed at advanced stages requiring additional systemic treatments. In such cases, tumor heterogeneity including presence of metastatic and/or chemo-resistant subclones is a major obstacle to cure the disease. The cancer stem cell model may give insights and bases to understand the tumor heterogeneity and to establish new strategies to treat them.

Cancer stem cells or tumor-initiating cells (TICs) are cells which possess the capacity to self-renew and to generate heterogeneous lineages of neoplastic cells that constitute the cancer [2]. TICs have been identified in many neoplasms, including tumors in the mammary gland [3], brain [4], prostate gland [5], colon [6], [7], pancreas [8], head and neck [9], and liver [10]. These TICs comprise about 1–5% of the whole tumor cells, and can form tumors again even when most cells are eliminated, for example, by chemotherapy. Thus it is important to identify gastric TICs and to characterize them to develop new therapies targeting them. There are several reports on the identification of gastric TICs, mostly using the cell surface marker CD44 [11]–[14]. A recent study demonstrated that CD44 played an important role in the tumorigenesis [15], but another study showed that CD44 was strongly expressed by both premalignant and malignant gastric epithelial cells, though it was rarely expressed in normal gastric mucosa [16]. Thus it remains to be examined whether CD44 is the best marker for gastric TICs.

In the present study, we could not obtain consistent results that CD44-positive gastric tumor cells were tumorigenic by analyzing patient-derived tumor xenograft (PDTX) cells. We thus looked for another marker for gastric TICs, and found that they strongly expressed CD49f, a subunit of laminin receptors, which has been used to identify TICs in tumors of the prostate gland [17], mammary gland [18], brain [19] and colon [20]. We established a primary culture system for PDTX cells where only CD49f^high^ cells could grow on extracellular matrix (ECM) to form ECM-attaching spheres, a feature of stem cells [21]. These CD49f^high^ sphere cells formed tumors with histological features of parental ones when injected into immunodeficient mice, indicating that only TICs could grow in culture. We also found that some CD49f^high^ sphere-forming TICs were more resistant to chemotherapeutic agents than gastric tumor cell lines, although there was a patient-dependent difference on their response. We thus conclude that CD49f is a promising marker for gastric TICs, and that this culture system will be useful to find new drugs targeting gastric TICs.

## Materials and Methods

### Tumor Tissues and PDTX Lines

Gastric tumor tissues were obtained with informed consent from patients who underwent surgical resection at Tokyo Medical and Dental University Hospital and Asan Medical Center Hospital between 2008 and 2012, and the study was approved by the Medical Research Ethics Committee for Genetic Research of Tokyo Medical and Dental University, and the Institutional Review Board of Asan Medical Center. Written informed consent was obtained from each patient for the use of his/her tumor tissue for this research in both hospitals. Freshly isolated tumor samples were cut into small pieces and transplanted subcutaneously into KSN and BALB/c nude mice at 4–6 weeks old (Japan SCL, Inc., Shizuoka, Japan and Central Lab. Animal Inc., Seoul, Korea, respectively). The animals were housed in specific pathogen-free animal facilities in accordance with the Guideline for Care and Use of Laboratory Animals of the respective Institutional Animal Care and Use Committees, and the research was approved by the Institutional Animal Care and Use Committee of Tokyo Medical and Dental University, and the Institutional Animal Care and Use Committee of Asan Medical Center. When gastric tumors grew in nude mice, some tissues were stored in liquid nitrogen, and were used at early passages (p<6) because tumors grew faster and became more aggressive with the increase in passage number. Gastric tumor tissues just after surgical operation were also used for the analysis.

### Dissociation and Staining of Tumor Cells for FACS Analysis

Tumor tissues were disaggregated into single cells for FACS (fluorescence-activated cell sorter) analysis. Tissues were cut with scalpels into small fragments, washed thoroughly with CMF-PBS, treated with 0.05% trypsin-0.53mM EDTA with 0.01% DNase I (Sigma-Aldrich, St Louis, MO, USA) for 30 min at 37°C, and cells were disaggregated by pipetting. In some previous papers, collagenases were used for cell dissociation, but we used trypsin-EDTA because it did not affect cell surface antigen profiles and reproducible results were easily obtained, as has been shown in a previous paper [11]. Cell suspensions were filtered through 70 µm nylon meshes (BD Biosciences, San Jose, CA, USA) to obtain single cells, which were stained with PE-labeled anti-human CD133/2 (clone AC133, Miltenyi Biotec, Auburn, CA, USA), PE- or FITC-labeled rat anti-CD44 (clone IM7, BD Biosciences), FITC-labeled rat anti-human CD49f (clone GoH3, BD Biosciences) or PE-labeled anti-EpCAM (clone HEA-125, Miltenyi Biotec) antibodies. Flow cytometric analysis and cell sorting were performed by using FACS Vantage or FACS Aria II cell sorters (BD Biosciences).

### Tumorigenicity Assay

Cells were counted and graded numbers of cells were suspended in 50 µl CMF-PBS. Cell suspension was mixed with an equal volume of BD Matrigel™ Basement Membrane Matrix (BD Biosciences) and subcutaneously injected into NOD-SCID mice (Charles River Japan, Yokohama, Japan and Central Lab. Animal Inc, Seoul, Korea) on the dorsal side of each flank. To minimize experimental variability due to individual differences in recipient mice, cell populations that were to be compared were injected on opposite flanks of the same animal. The injected mice were maintained for up to 6 months and killed when tumor diameters reached 10 mm.

### Histological Analysis

Tumor tissues were fixed with 95% ethyl alcohol, embedded in paraffin and sectioned at 5 µm. Sections were deparaffinized and hydrated using xylene and ethyl alcohol, and stained with hematoxylin and eosin, and Alcian blue (pH2.5)-PAS-hematoxylin. For immunohistochemical detection of CD49f in gastric tissues, an automatic staining device (Benchmark XT; Ventana Medical Systems, Tucson, AZ, USA) was used. Cryostat sections fixed with acetone were treated with rat anti-CD49f monoclonal antibody (Clone NKI-GoH3, Merck Millipore, Billerica, MA, USA), followed by a biotinylated anti-mouse immunoglobulin and peroxidase-labeled streptavidin (LSAB kit; DAKO, Carpinteria, CA, USA). The localization of the antigen was visualized by using 3,3′-diaminobenzidine as chromogen. Harris’ hematoxylin was used to show the tissue structure.

### Cell Culture

Tumor samples were dissociated into single cells as described above. The resulting cells were cultured on rat tail collagen gel or Matrigel in a serum-free condition. We have previously established a primary culture system for rat gastric epithelial cells [22], where we used F-12 medium supplemented with EGF, insulin, hydrocortisone, transferrin, and cholera toxin. We found in the present study that some human gastric tumor cells grew slowly in the medium. We thus compared their growth in various media for normal human epithelial cells provided from Lonza (Basel, Switzerland), and found that cells grew best in REBM medium which contains EGF, insulin, hydrocortisone, transferrin, triiodothyronine, and epinephrine. In the present study, cells were cultured in REBM medium with B27 supplement (Invitrogen, Carlsbad, CA, USA), FGF-2 (ReproCELL, Kanagawa, Japan), Y-27632 ROCK inhibitor (Merck Millipore) and gastrin (Sigma-Aldrich), by modifying the method developed for mouse gastric epithelial cells [23].

### RT-PCR

The gene expression profiles of gastric cancer cells were examined by RT-PCR. Total RNAs were extracted from cells or tissues by using the Allprep DNA/RNA/Protein Mini kit (Qiagen, Valencia, CA, USA). RNAs were resuspended in RNase-free water and first-strand cDNAs were synthesized by using the SuperScript III First-Strand Synthesis SuperMix (Invitrogen). The primer sequences and PCR conditions are described in Table S1. *GAPDH* was used as a control to compensate for varying efficiencies of extraction and reverse transcription. The PCR products were separated by electrophoresis on 2.0% agarose gels in 0.5×TAE buffer.

### Effect of Chemotherapeutic Agents on the Growth of TICs in Primary Culture

HGC-1, HGC-2 and HGC-4 tumors were dissociated into single cells as described above, and seeded on collagen gel in 24-well plates at 50,000 cells per well with REBM-based culture medium, and their growth was examined after 2 weeks. MKN45 and MKN74 human gastric cancer cells were maintained in RPMI1640 medium supplemented with 10% fetal bovine serum on plastic, but we found that both cells proliferated rapidly in the REBM-based serum-free condition used for culture of HGC-1, HGC-2 and HGC-4 cells. We thus used both cells as controls. Since MKN45 cells proliferated more rapidly than MKN74 cells, we seeded MKN45 cells more sparsely (1,000 cells per well) than MKN74 cells (3,000 cells per well) to give enough space for the cells to keep growing for 2 weeks.

The cells were plated with 0.5 ml media on day 0, and they were treated with doxorubicin (DXR), 5-fluorouracil (5-FU), and doxifluridine (DXF) (Wako Pure Chemical, Osaka, Japan) on day 1, by adding 0.5 ml media containing ×2 concentrated chemicals. 5-FU and DXF were dissolved in dimethyl sulfoxide (DMSO), while DXR in water. Thus DMSO (final concentration = 0.1%) was added to each well when cells were treated with 5-FU or DXF. After 2 weeks, cell growth was determined by using 3-(4,5-di-methylthiazol-2-yl)-2,5-diphenyltetrazolium bromide (MTT) as substrate as described previously [24].

## Statistical Analyses

The results were statistically analyzed using the non-parametric Mann-Whitney’s *U* test or Student’s *t-*test. For the analysis of data in tables, Chi-square test was used. A *P* values of <0.05 were considered statistically significant.

## Results

### CD49f is a useful Marker for Gastric TICs

We first established PDTX lines by subcutaneously injecting newly-dissected human gastric tumor tissues into nude mice, because such lines have been used to identify TICs in colon [6], [25] and lung [26] cancers. We established 5 PDTX lines (HGC-1 to -5, Table 1), and confirmed that histological features of parental tumors were maintained in PDTXs even when tissues were passaged several times (data not shown).

**Table 1:** Case description and tumorigenic activity of CD49f^high^ and CD49f^low^ tumor cells.

Patientnumber	Age/sex	Site	Bormann type	Differentiationof tumors	PDTX lines	Ratio of CD49^high^cells (%)	Number of cells injected (number oftumors/number of injection)		Differentiation of tumors formed by sorted cells
							CD49f^high^	CD49f^low^	
1	80/M	Fundic	II	Moderate	HGC-1	2.7	3,000 (1/1)	6,000 (0/1)	Moderate
2	75/M	Fundic	III	Well	HGC-2	6.5	3,000 (3/3)	3,000 (1/2)	Well
3	72/M	Pyloric	IV	Poor	HGC-3	16.4	3,000 (1/1)	3,000 (0/2)	Poor
4	71/M	Fundic	II	Moderate	HGC-4	6.2	3,000 (1/1)	3,000 (0/1)	Moderate
5	68/F	Pyloric	II	Poor	HGC-5	10.8	3,000 (3/3)	3,000 (1/4)	Poor
6	66/M	Pyloric	II	Moderate		14.8	3,000 (1/1)	3,000 (0/1)	Moderate
7	74/M	Antrum	III	Poor		20.8	3,000 (1/1)	3,000 (1/1)	Poor
8	64/M	Antrum	II	Moderate		8.8	3,000 (1/2)	3,000 (0/1)	Moderate
9	40/M	Fundic	III	Poor		5.3	3,000 (1/1)	3,000 (0/1)	Poor
10	72/M	Antrum	III	Poor		3.0	1,000 (1/1)3,000 (1/1)	1,000 (0/1)3,000 (0/1)	Poor
11	70/M	Fundic	II	Moderate		10.9	3,000 (1/1)	3,000 (0/1)	Moderate
12	68/M	Antrum	III	Poor		1.1	3,000 (1/1)	3,000 (0/1)	Poor
13	68/M	Antrum	II	Poor		1.6	3,000 (1/2)	3,000 (0/2)	Poor
14	76/F	Pyloric	III	Poor		2.8	3,000 (2/2)	3,000 (0/2)	Poor
15	71/M	Pyloric	III	Moderate		5.5	3,000 (1/2)	3,000 (0/2)	Moderate
Total					5	7.9±6.1	3,000 (20/23)***	3,000 (3/22)***	

*** P<0.001 by Chi-square test.

CD44 has been reported as a marker for gastric TICs [11]–[14]. We thus compared tumorigenicity of CD44-positive and -negative cells in the PDTXs, and found that not only CD44^high^ cells but also CD44^low^ cells were tumorigenic in 3 PDTX lines (Figure S1 and Table S2). Rocco et al. [27] also reported that CD133^+^ and CD133^+^/CD44^+^ cells in human primary gastric cancers did not exhibit tumor-initiating activities when they were transplanted into immunodeficient mice. These results suggest that TICs in primary gastric cancers do not always express CD44. We thus looked for another marker which was specifically expressed by gastric TICs.

We found a significant difference in tumorigenicity between CD49f^high^ and CD49f^low^ cells. CD49f^high^ cells very frequently formed tumors with histological features of parental ones while CD49f^low^ cells were not tumorigenic (HGC-1 to -5, Table 1). Moreover, cell surface antigen profiles were maintained in tumors formed by injection of CD49f^high^ cells (Figure S2), indicating that CD49f^high^ cells retained self-renewal and differentiation potencies.

**Figure 1:**
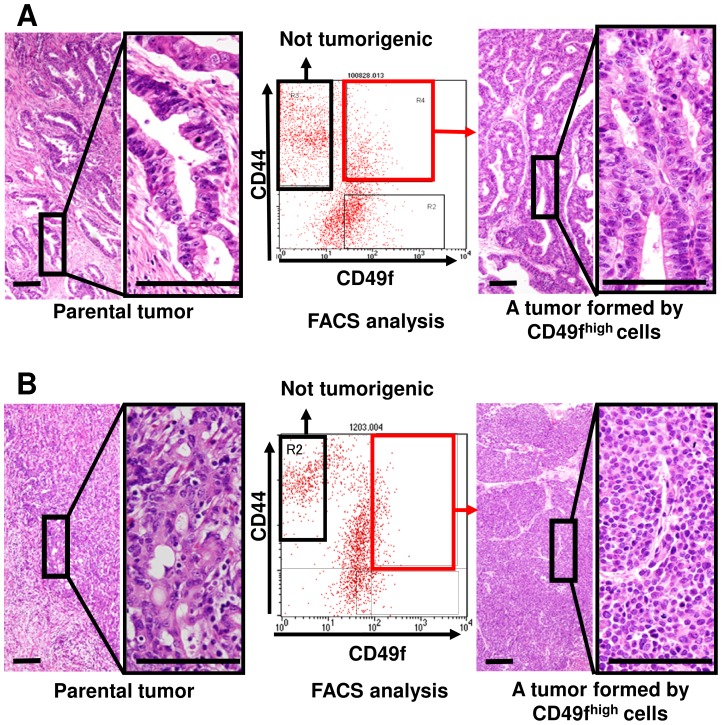
CD49f^high^ cells form tumors while CD49f^low^ cells do not. (A) A moderately-differentiated adenocarcinoma from patient #8 and (B) a poorly-differentiated one from patient #9 in Table 1 were dissociated, and FACS-sorted cells were subcutaneously injected into NOD-SCID mice to examine their tumorigenicity. Histological features of parental tumors (left) correspond well with those of tumors (right) formed by injection of sorted cells (shown by red rectangles in FACS analysis). Tissue specimens are stained with hematoxylin and eosin. Scale bars represent 100 µm.

We found that the cell surface antigen profiles of PDTX cells (HGC-1 to -5) were altered when passage number exceeded 12 (data not shown), possibly because highly tumorigenic subpopulations were selected during in vivo passages, as has been reported in pancreatic cancer [28]. We thus examined whether CD49f^high^ cells were more tumorigenic than CD49f^low^ cells using newly-dissected gastric tumors (HGC-6 to -15, Table 1). We found that CD49f^high^ cells always formed tumors with histological features of parental ones, while CD49f^low^ cells were not tumorigenic, independent of the tumor type (Table 1 and Figure 1: Figure 1A for a moderately-differentiated adenocarcinoma from patient #8 and Figure 1B for a poorly-differentiated one from patient #9). There was a significant difference between CD49f^high^ and CD49f^low^ cells on their tumorigenicity (Table 1). We thus concluded that CD49f is a useful marker for gastric TICs.

### CD49f is Localized at the Epithelial-stromal Interface in the Normal Gastric Mucosa, but is Found on Apical, Lateral and Basal Surfaces in Some Tumor Cells

**Figure 2:**
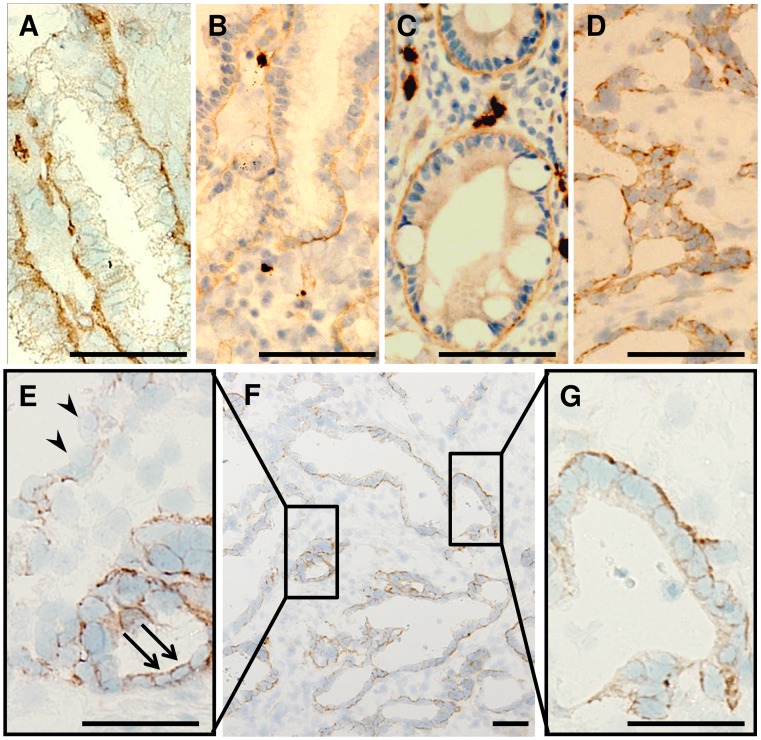
Localization of CD49f in gastric tissues. (A–C) CD49f is localized at the epithelial-stromal interface in (A) normal mucosa, (B) chronic gastritis and (C) intestinal metaplasia. (D–G) In (D) gastric dysplasia adjacent to carcinoma and (E–G) gastric tumor tissues, expression of CD49f is diversified: It is localized at the epithelial-stromal interface in some cells (G), but on apical, lateral and basal surfaces in some cells (E, arrows), and expression can hardly be detected in some cells (E, arrowheads). Scale bars represent 50 µm.

We immunohistochemically examined the localization of CD49f in the tumor and non-tumor tissues in the stomach, and found CD49f at the epithelial-stromal interface in the normal gastric mucosa (Figure 2A). The result is consistent with the report that CD49f functions as a subunit of receptors for laminins, major proteins in the basal lamina. Gastric mucosae with chronic gastritis showed expression of CD49f along the basal lamina of the gastric foveolae as in normal ones (Figure 2B), and similar expression pattern was found in the intestinal metaplasia (Figure 2C). In tumor cells, however, expression of CD49f was diversified. It was found at the epithelial-stromal interface in some tumor cells (Figure 2G), but it was detected on apical, lateral and basal surfaces in some cells, and some cells only weakly expressed it (Figure 2E). It is interesting that in gastric dysplasias adjacent to carcinoma, CD49f was found on not only basal but also lateral surfaces, as in gastric carcinomas (Figure 2D), indicating that disorganized expression of CD49f might not be a result but a cause of tumorigenesis. A small proportion of tumor cells expressing CD49f all along the cell membrane (Figure 2E) may possibly represent gastric TICs.

### CD49f^high^ Cells Attach to ECM to form Spheres in Primary Culture

TICs have been reported to grow in serum-free conditions to form spheres in several solid tumors, and sphere formation assay is widely used to detect TICs [21], [29]. We thus cultivated gastric tumor cells by modifying single cell culture system for mouse gastric epithelial cells [23]. We found that ECM (collagen gel or Matrigel) was necessary for HGC-1, HGC-2 and HGC-4 PDTX cells to grow, and that they exhibited sphere-formation, a feature of stem cells [21] in culture. We thus used these PDTXs at early passages (P<6) to investigate further the relationship among CD49f expression, sphere formation and tumorigenesis.

**Figure 3:**
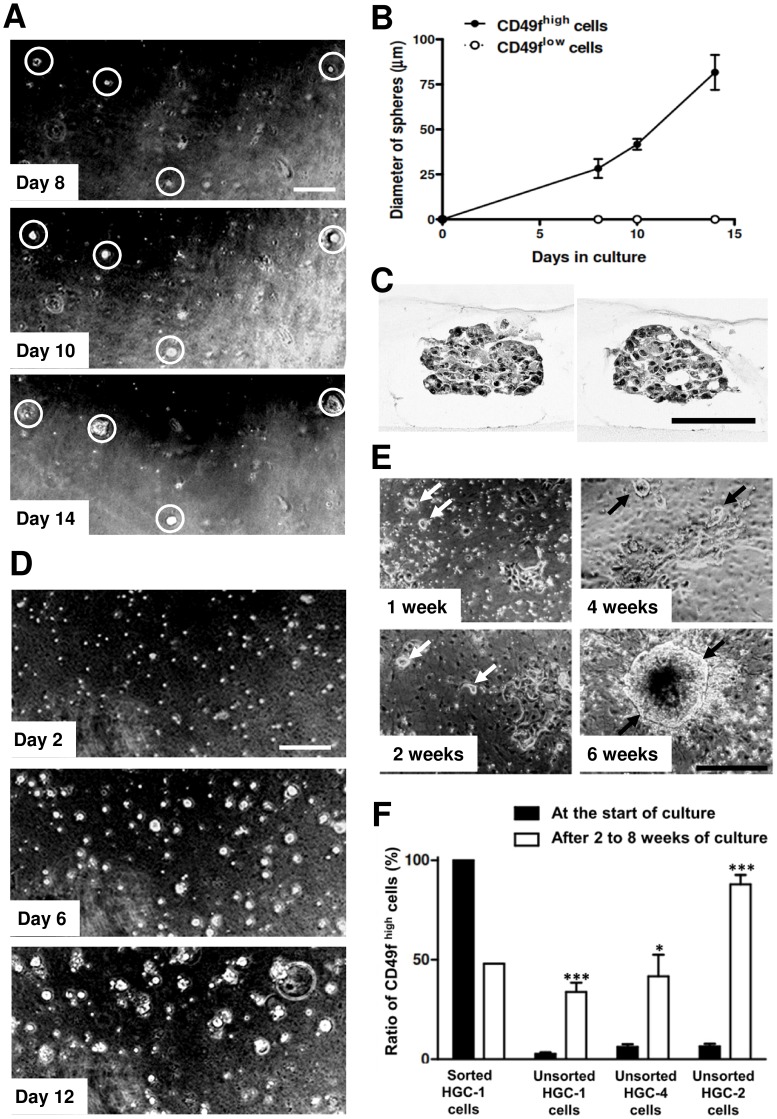
CD49f^high^ cells form spheres in primary culture. (A) Phase contrast micrographs showing the growth of spheres in culture of CD49f^high^ HGC-1 tumor cells on collagen gel. The same area on days 8, 10 and 14 are shown. It is clear that spheres in the white circles grow rapidly in culture. Scale bar represents 200 µm. (B) The increase in diameters of spheres in culture of CD49f^high^ HGC-1 tumor cells, shown in (A). (C) Light micrographs of spheres formed by culture of CD49f^high^ HGC-1 tumor cells for 2 weeks. Tissue specimens are stained with PAS-hematoxylin. Scale bar represents 100 µm. (D) Phase contrast micrographs showing the growth of spheres in culture of unsorted HGC-4 tumor cells on collagen gel. The same area on days 2, 6, and 12 are shown. Scale bar represents 200 µm. (E) Phase contrast micrographs showing the growth of spheres in culture of unsorted HGC-2 tumor cells on collagen gel. Many flat cells closely attach to ECM to form a thin layer. Sphere-forming cells (arrows) grow slowly on the flat cells to form large spheres at 6 weeks. Scale bar represents 500 µm. (F) Changes in the ratio of CD49f^high^ cells in total cells in culture of sorted CD49f^high^ HGC-1 tumor cells (for 2 weeks), unsorted HGC-1 tumor cells (for 2 weeks), unsorted HGC-4 tumor cells (for 2 weeks) and unsorted HGC-2 tumor cells (for 8 weeks). Significant increases in the ratio of CD49f^high^ cells are always found in culture of unsorted tumor cells. ***, P<0.001; *, P<0.05 by Student’s t-test.

When HGC-1 gastric tumor cells were cultured after FACS-sorting, the cloning efficiency was usually less than 10%, but CD49f^high^ cells attached to collagen gel to form spheres (Figure 3A), whereas cell growth was not observed in culture of CD49f^low^ cells (Figure 3B). These spheres were consisted of epithelial cells closely attached to each other with occasional PAS-positive droplets (Figure 3C). It was difficult to cultivate FACS-sorted HGC-2 and HGC-4 cells, and we were not certain whether only CD49f^high^ cells could form spheres in their culture. We thus concluded that some gastric TICs proliferated to form spheres in primary culture as has been reported in other TICs.

### Only CD49f^high^ Sphere-forming TICs can Grow when Unsorted Tumor Cells are Cultured

One possible reason why FACS-sorted HGC-2 and HGC-4 cells did not grow in culture is that they may need autocrine factors to grow, and their concentrations may be less than the threshold value when cells were sparsely cultured (usually we cultured FACS-sorted cells at 10 cells/mm^2^, because of technical reasons). If this hypothesis is correct, cells may grow when cultured closely. We thus tried to cultivate tumor cells much more closely (usually 300 cells/mm^2^) by seeding them without FACS-sorting. When dissociated HGC-1 and HGC-4 tumor cells were cultured on collagen gel without FACS-sorting, many cells formed spheres, whose diameter increased greatly in 2 weeks (Figure 3D), consistent with the above assumption. When dissociated HGC-2 cells were cultured without FACS-sorting on Matrigel or collagen gel, some HGC-2 cells attached closely to ECM to form a thin layer, and some cells formed spheres on the cell layer. In culture of HGC-2 cells, some flat cells slowly proliferated in the first 2 weeks, but they stopped growth soon, while sphere-forming cells kept growing to form large spheres after 6 to 8 weeks (Figure 3E). Thus the growth pattern of HGC-2 tumor cells was different from that of HGC-1 and HGC-4 cells, though all three tumor cells were similar in that ECM was necessary for the tumor cells to grow to form spheres in culture. We examined features of HGC-2 sphere cells at 8 weeks when pure sphere-forming cells could be easily obtained.

When FACS-sorted CD49f^high^ HGC-1 cells were cultured for 2 weeks, the ratio of CD49f^high^ cells was decreased to about 50% (Figure 3F), indicating that CD49f^high^ cells differentiated into both CD49f^high^ and CD49f^low^ cells in vitro. When unsorted HGC-1 cells were cultured, the ratio of CD49f^high^ cells was significantly increased about 10-times from 3 to 34%. Also it was increased about 7-times from 6 to 42% in culture of unsorted HGC-4 cells in 2 weeks (Figure 3F). The ratio of CD49f^high^ cells in total cells was similar when sorted CD49f^high^ HGC-1 cells or unsorted (mostly CD49f^low^) HGC-1 cells were cultured. This indicates that only CD49f^high^ cells could grow to form spheres when unsorted HGC-1 tumor cells were cultured. In culture of unsorted HGC-2 tumor cells, the ratio of CD49f^high^ cells was significantly increased more than 10-times from 6 to 88% in 8 weeks. Thus all these tumor cells retain similar features that only sphere-forming CD49f^high^ cells could grow in vitro, but their growth pattern differed depending on PDTX lines. These sphere-forming cells as well as PDTXs strongly expressed *BMI1, POU5F1 and SOX2* (Figure S3), which are reported to be strongly expressed by stem cells [30].

**Figure 4:**
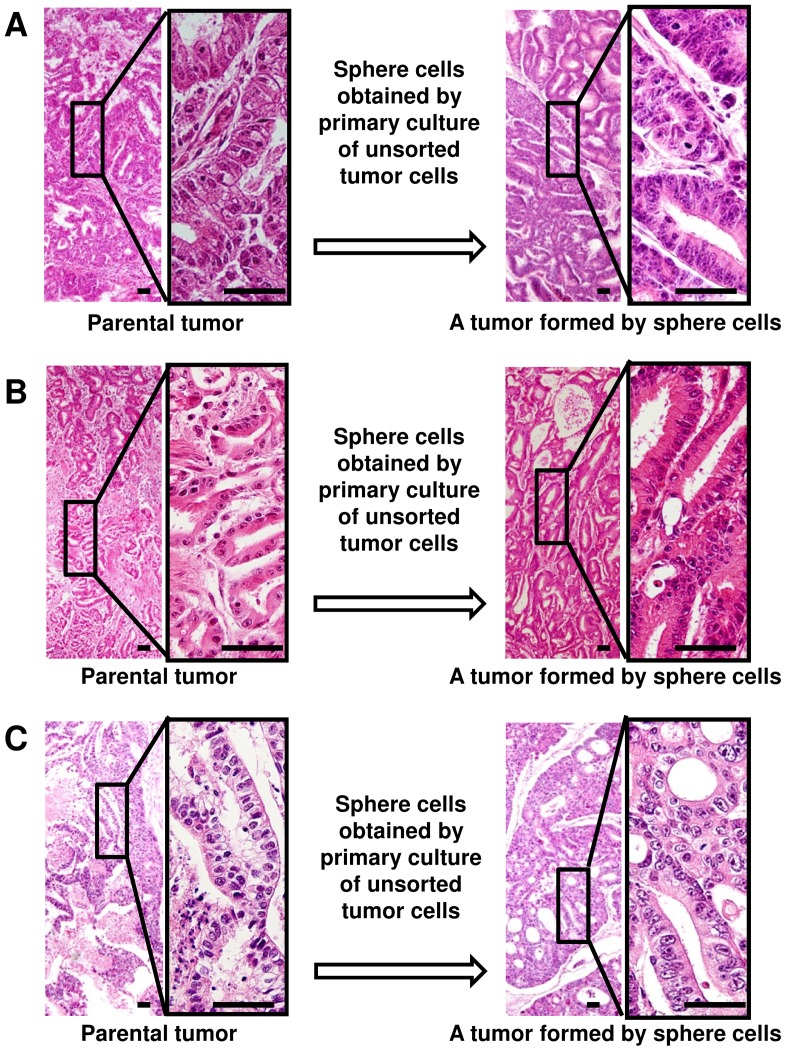
Sphere cells form tumors with histological features of parental ones. Light micrographs of tumors formed by subcutaneous injection of sphere cells into NOD-SCID mice (right panels), which are obtained by cultures of (A) unsorted HGC-1, (B) unsorted HGC-2 and (C) unsorted HGC-4 tumor cells. Histological features of tumors formed in mice correspond well with those of parental ones (left panels). Tissue specimens are stained with hematoxylin and eosin. Scale bars represent 50 µm.

We then examined tumorigenicity of these sphere-forming CD49f^high^ cells. We found that sphere cells obtained by primary culture of unsorted HGC-1 tumor cells were strongly tumorigenic because subcutaneously injection of 3,000 sphere cells always formed tumors and injection of 10 cells sometimes formed a tumor in NOD-SCID mice (Table 2). The tumors exhibited histological features of the parental one (Figure 4), indicating that the CD49f^high^ sphere-forming cells retained differentiation potency of TICs in the original tumor. Sphere cells obtained by culture of unsorted HGC-2 and HGC-4 cells also formed tumors with histological features of parental ones (Table 2 and Figure 4), but tumorigenicity of HGC-4 sphere cells was less than that of HGC-1 sphere cells, and that of HGC-2 sphere cells was at the intermediate between HGC-1 and HGC-4 sphere cells (Table 2). We thus concluded that only CD49f^high^ sphere-forming TICs could grow when unsorted tumor cells were cultured. These results are consistent with our idea that CD49f is a useful marker for gastric TICs.

**Table 2:** Tumorigenicity of sphere cells obtained by culture of unsorted cells (number of tumors formed in NOD-SCID mice/number of injection of tumor cells).

PDTX line	Number of injected cells					
	3,000	1,000	300	100	30	10
HGC-1	8/8**	4/8	5/10*	1/6	0/2	1/2
HGC-2	3/4	3/4	2/4	0/2	0/2	0/2
HGC-4	2/6**	1/6	0/6*	0/6	0/4	–

** P<0.01;

* P<0.05 by Chi-square test;

–not determined.

### Some Sphere-forming TICs are Resistant to Chemotherapeutic Agents

TICs have been reported to be more resistant to chemotherapy than other tumor cells [31]. We thus examined whether the CD49f^high^ sphere-forming TICs were resistant to anti-tumor drugs. We found that MKN45 and MKN74 human gastric tumor cell lines attached to the collagen gel to grow rapidly in the same condition where unsorted HGC-1, HGC-2 and HGC-4 tumor cells grew to form spheres. We thus used MKN45 and MKN74 cells as controls.

**Figure 5:**
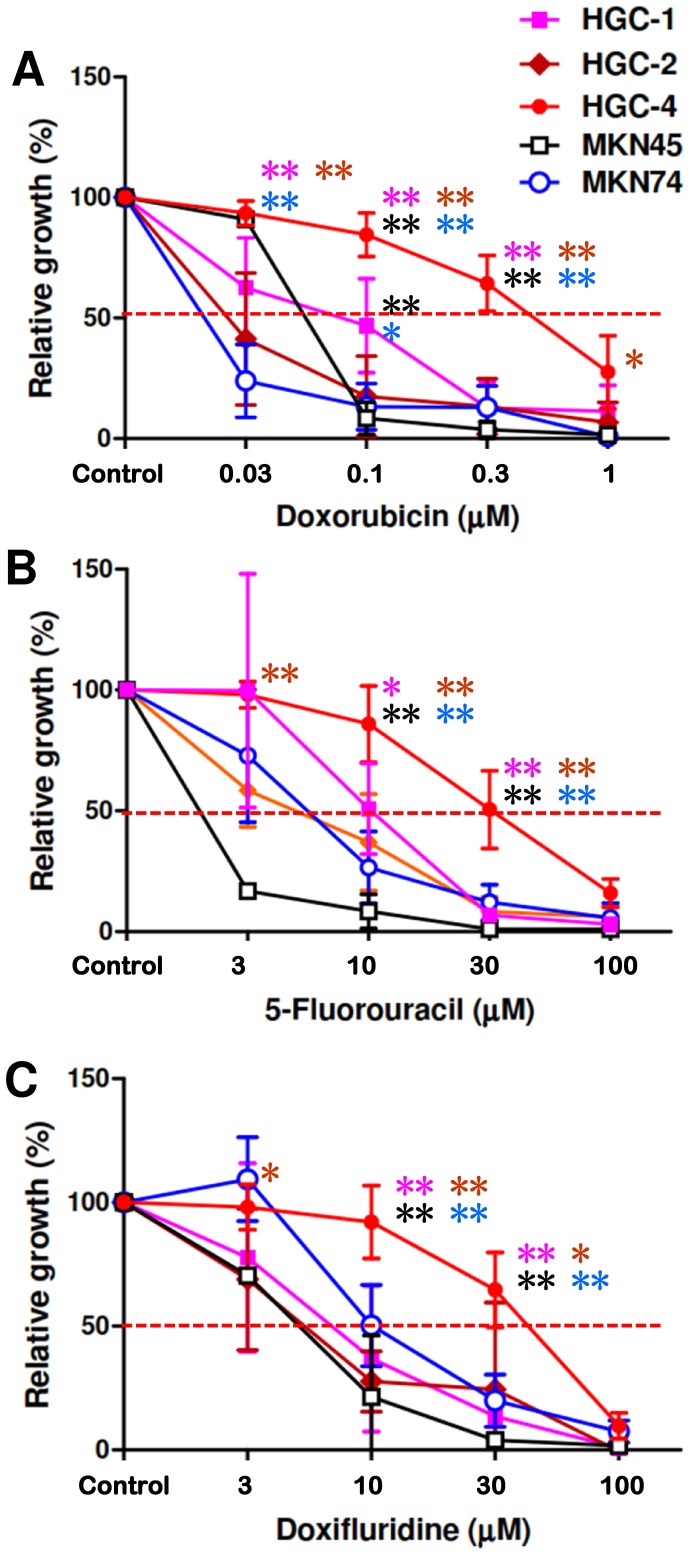
Some TICs are more resistant to anti-tumor drugs than gastric tumor cell lines and other TICs. HGC-1, HGC-2 and HGC-4 tumor cells, and MKN45 and MKN74 tumor cell lines are seeded on day 0, treated with various concentrations of (A) doxorubicin, (B) 5-fluorouracil, and (C) doxifluridine from days 1 to 14, and cell numbers on day 14 are determined by MTT assay. Black and blue asterisks show that HGC-1 and/or HGC-4 cells are significantly (**, P<0.01; *, P<0.05 by Student’s t-test) more resistant to the drugs than MKN45 and MKN74 cell lines, respectively, while pink and brown asterisks indicate that HGC-4 cells are significantly (**, P<0.01; *, P<0.05 by Student’s t-test) more resistant to the drugs than HGC-1 and HGC-2 cells, respectively.

We found that unsorted HGC-1 and HGC-4 tumor cells formed spheres in the presence of anti-tumor drugs. It seemed that not the sphere formation but the growth of spheres was suppressed by the drugs because many small spheres were found when HGC-1 or HGC-4 tumor cells were treated with drugs at higher concentrations (Figure S4). MKN45 and MKN74 cells never formed spheres when cultured in a condition where HGC-1 and HGC-4 cells formed spheres, indicating that sphere formation could not be induced in these cell lines (Figure S4). The growth of MKN45 and MKN74 cells was severely affected by the drugs, with EC_50_ (half maximal effective concentration) of 0.02–0.05 µM for DXR, 2–6 µM for 5-FU and 4–10 µM for DXF (Figure 5). In contrast, growth of HGC-4 cells was less affected by them, with EC_50_ of 0.5 µM for DXR, 30 µM for 5-FU and 40 µM for DXF (Figure 5). This indicates that HGC-4 cells were far more resistant than gastric tumor cell lines to anti-tumor agents. HGC-1 and HGC-2 cells were similar to gastric tumor cell lines concerning their responses to the drugs, though HGC-1 cells were more resistant to DXR than tumor cell lines at 0.1 µM. In culture of unsorted HGC-2 cells, flat cells were major in the first 2 weeks (Figure 3E). Thus it was difficult to examine the response of sphere-forming HGC-2 cells to the drugs by examining the cell number at 2 weeks in culture. It is possible that sphere-forming HGC-2 cells may be more resistant to the drugs than gastric tumor cell lines, if another culture system with longer cultivation period (eg. 6–8 weeks) is used for the analysis. HGC-4 cells were always more resistant to the drugs than HGC-1 and HGC-2 cells, suggesting that there are significant patient-dependent differences between gastric TICs on their responses to chemotherapeutic agents.

## Discussion

In the present study, we found that human gastric TICs strongly expressed CD49f on their surface, using 15 primary gastric carcinoma cases. Previously, gastric TICs have been identified by using CD44 [11], CD44 and EpCAM [12], CD44 and CD24 [13], CD44 and CD54 [14], CD90 [32], and aldehyde dehydrogenase 1 [33] as markers, but CD49f has not been used to detect TICs in gastric cancers. This may be the first report showing that CD49f is a promising marker for gastric TICs. We then established a primary serum-free culture system for them, where only CD49f^high^ cells could grow to form ECM-attaching spheres with strong tumorigenicity.

CD49f or integrin α6 (*ITGA6*) is a 150 kDa transmembrane protein, expressed mainly on T cells, monocytes, and epithelial and endothelial cells [34]. CD49f associates with integrin β1 chain (CD29) to form VLA-6, and with integrin β4 chain (CD104) to form the α6β4 complex, both of which are known to function as the laminin receptor [35]. An emerging consensus is that α6β4 complex synergizes with specific molecules such as ErbB2, EGFR, Ron, Fyn, c-Met, protein kinase C, CD151, and CD9 to activate key signaling pathways for the invasion and migration of carcinoma cells via activation of signaling molecules such as PI3K [36], [37]. In previous experiments with mammary and brain tumor cells [18], [19], CD49f^high^ cells were cultured in non-adherent conditions to induce sphere formation. Thus attachment to ECM may not be necessary for some CD49f^high^ cells to exhibit tumorigenic activity while other CD49f^high^ cells were cultured just in the presence of ECM [17], [38]. In previous studies with gastric TICs, cells were cultured on non-adherent substrata to form floating spheres [12]–[14], [32], and substratum was used to induce their differentiation into non-tumorigenic cells. In the present study, we found that CD49f^high^ cells could not grow on non-adherent substrata, and they could survive only in the presence of ECM in all three cases analyzed (data not shown). This suggests that characteristics may be different between TICs identified in the present study and those reported in previous ones though both formed spheres in vitro and exhibited strong tumorigenicity in vivo.

It is well known that growth, differentiation and progression of cancer cells are severely affected by ECM [39]. The role of laminin on the progression of tumors has been intensively investigated. Woo Ho Kim, one of co-authors, has repeatedly reported in collaboration with Hynda Kleinman that laminin-adherent human colon cancer cells exhibited strong tumorigenicity, increased growth and decreased apoptosis [40], [41], and similar results have been reported in pancreatic cancers [42]. Consistent with these, gastric carcinomas have been reported to use α6β4 integrin and newly deposited laminins to adhere to surrounding tissues during the invasion [43], and Koike et al. [44] showed that invasive behavior of gastric cancer cells was inhibited by treatment with anti-α6 integrin antibody. These results strongly indicate that CD49f plays an important role in regulating invasiveness of human gastric cancers, but its molecular mechanism remains to be solved. Recently, Yu et al. reported that OCT4 and SOX2 directly bind to the *ITGA6* promoter to induce the expression of CD49f, and that CD49f plays a pivotal role in maintaining cellular pluripotency through the PI3K/AKT/p53 pathway in mesenchymal stem cells [45]. Consistent with this, we found that *SOX2*, *POU5F1* (a gene encoding OCT4) and *ITGA6* were all strongly expressed in human gastric tumors examined, except that *SOX2* was not expressed by MKN74 cell line (Figure S3). Our culture system for CD49f^high^ gastric TICs will be useful for further analysis on the role of CD49f in gastric tumorigenesis.

We found in the present investigation that CD49f^high^ gastric TICs formed ECM-attaching spheres. There are reports showing that substrata are essential to induce sphere-formation by TICs in brain [46], [47] and prostate gland [17], [38], [48] tumors while others show that TICs can form spheres in non-adherent conditions, as described above. This indicates that TICs may be divided into two types, depending on whether ECM is needed for their growth or not. It is well known that embryonic stem cells and induced pluripotent stem cells need feeder cells/ECM including laminins for their survival and growth in vitro [49]. We have also demonstrated that normal gastric epithelial cells can survive only in the presence of ECM [50]. Thus gastric TICs identified in the present study seem to retain some characteristics of normal stem cells, and TICs which can grow in the non-adherent condition may be derived from those which need ECM to grow. Our culture system may be useful to analyze how ECM-independent TICs emerge from ECM-dependent ones, and how it is related to the progression of cancer.

It is interesting that HGC-1 cells were more tumorigenic than HGC-4 cells (Table 2), while the latter was more resistant to chemotherapeutic agents than the former (Figure 5), indicating that chemo-resistance and tumorigenicity are independent features of TICs. Recently several drugs have been identified which specifically suppress the growth and induce the differentiation/apoptosis of TICs [51]–[53]. Our culture system may also be useful to identify such new drugs that selectively target gastric TICs, especially at the first stage of tumorigenesis. Such compounds may work as prophylactic drugs to suppress gastric cancer especially for individuals and families at high risk for the disease.

## Supporting Information

Figure S1 **CD44-negative, CD133-negative cells form tumors with histological features of parental ones.** HGC-2 and HGC-5 PDTXs were dissociated into single cells, and their tumorigenicity was analyzed by injecting sorted cells into immunodeficient mice. CD44-negative, CD133-negative cell fractions (shown by red squares in FACS analyses) formed tumors with histological features of parental ones while CD44-positive cells (shown by black squares) were not tumorigenic. Tissue specimens are stained with Alcian blue-PAS-hematoxylin. Scale bars represent 50 µm.
(TIF)

Figure S2 **Cell surface antigen profiles are maintained in tumors formed by injection of CD49f^high^ cells.** Cell surface antigen profiles of HGC-3 and HGC-4 PDTX cells differed greatly, but these profiles of secondary tumors (right panels) formed by injection of CD49f^high^ cells (shown by red squares in FACS analyses) into immunodeficient mice were similar to those of primary tumors (left panels).
(TIF)

Figure S3 **Gene expression profiles of human gastric tumor cell lines, PDTXs and sphere-forming TICs.** MKN74 and MKN45 human gastric tumor cell lines, HGC-1 and HGC-4 PDTXs, and HGC-1 and HGC-4 sphere cells formed by culture of unsorted cells expressed stem cell-related genes including *BMI1, NANOG, POU5F1, SOX2* and *ITGA6* at similar levels though MKN74 cells did not express *SOX2*, and HGC-4 sphere cells expressed *NANOG* strongly.
(TIF)

Figure S4 **Phase contrast micrographs of doxorubicin (DXR)-treated HGC-1 and HGC-4 tumor cells, MKN45 and MKN74 tumor cell lines on day 14 in vitro.** Their growth was quantified by MTT assay and results are shown in Figure 5A. Scale bars represent 200 µm.
(TIF)

Table S1 Primer sequences and PCR conditions.
(DOCX)

Table S2 Case description and tumorigenic activity of CD44^high^ and CD44^low^ gastric cancer cells.
(DOCX)
