# Supplementary figures and images for: CD49fhigh Cells Retain Sphere-Forming and Tumor-Initiating Activities in Human Gastric Tumors
Source: PLoS One. 2013 Aug 28;8(8):e72438. doi: 10.1371/journal.pone.0072438 (PMC3756075; doi:10.1371/journal.pone.0072438)

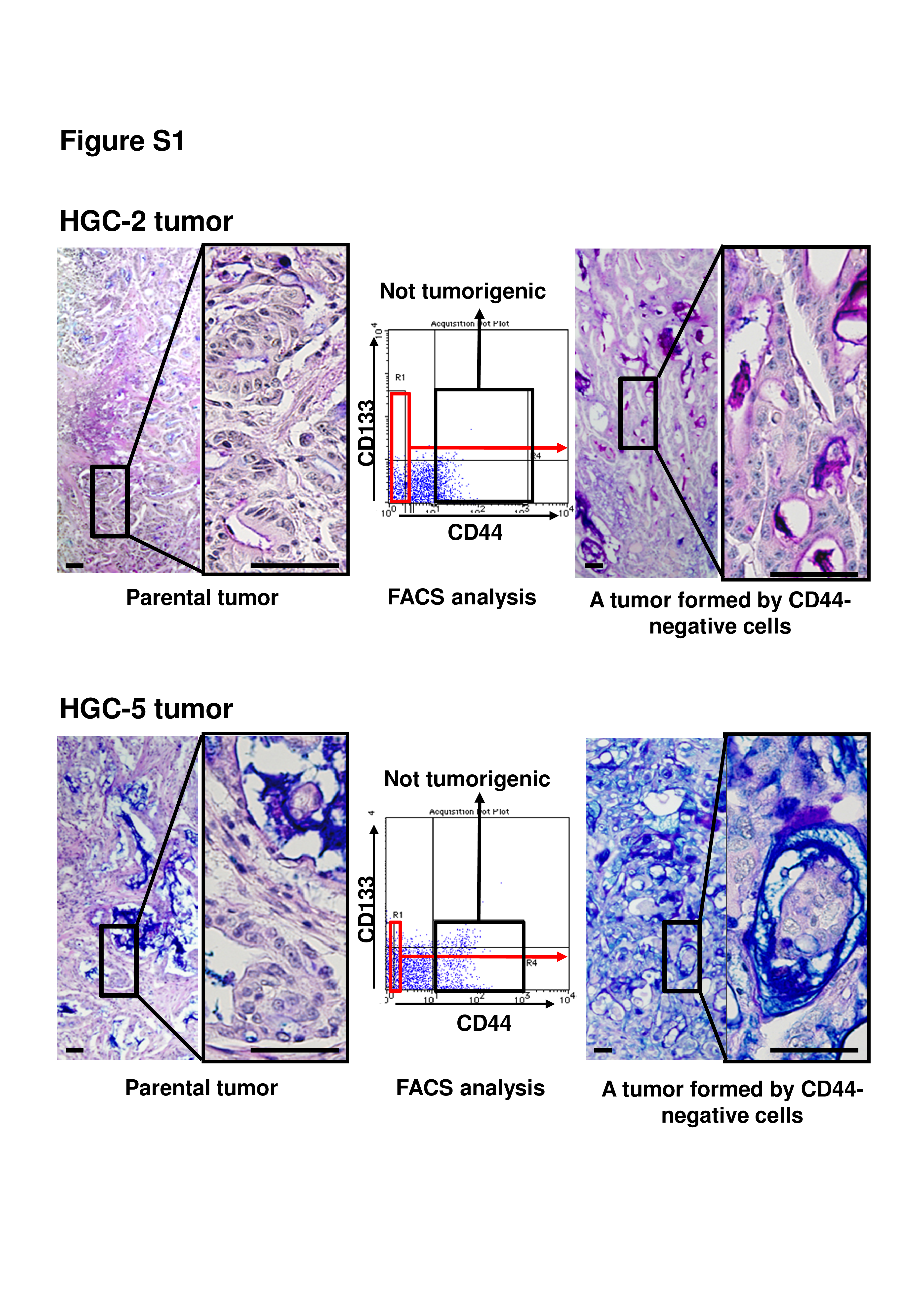

Supplement: Figure S1 — CD44-negative, CD133-negative cells form tumors with histological features of parental ones. HGC-2 and HGC-5 PDTXs were dissociated into single cells, and their tumorigenicity was analyzed by injecting sorted cells into immunodeficient mice. CD44-negative, CD133-negative cell fractions (shown by red squares in FACS analyses) formed tumors with histological features of parental ones while CD44-positive cells (shown by black squares) were not tumorigenic. Tissue specimens are stained with Alcian blue-PAS-hematoxylin. Scale bars represent 50 µm. (TIF) [file pone.0072438.s001.tif]

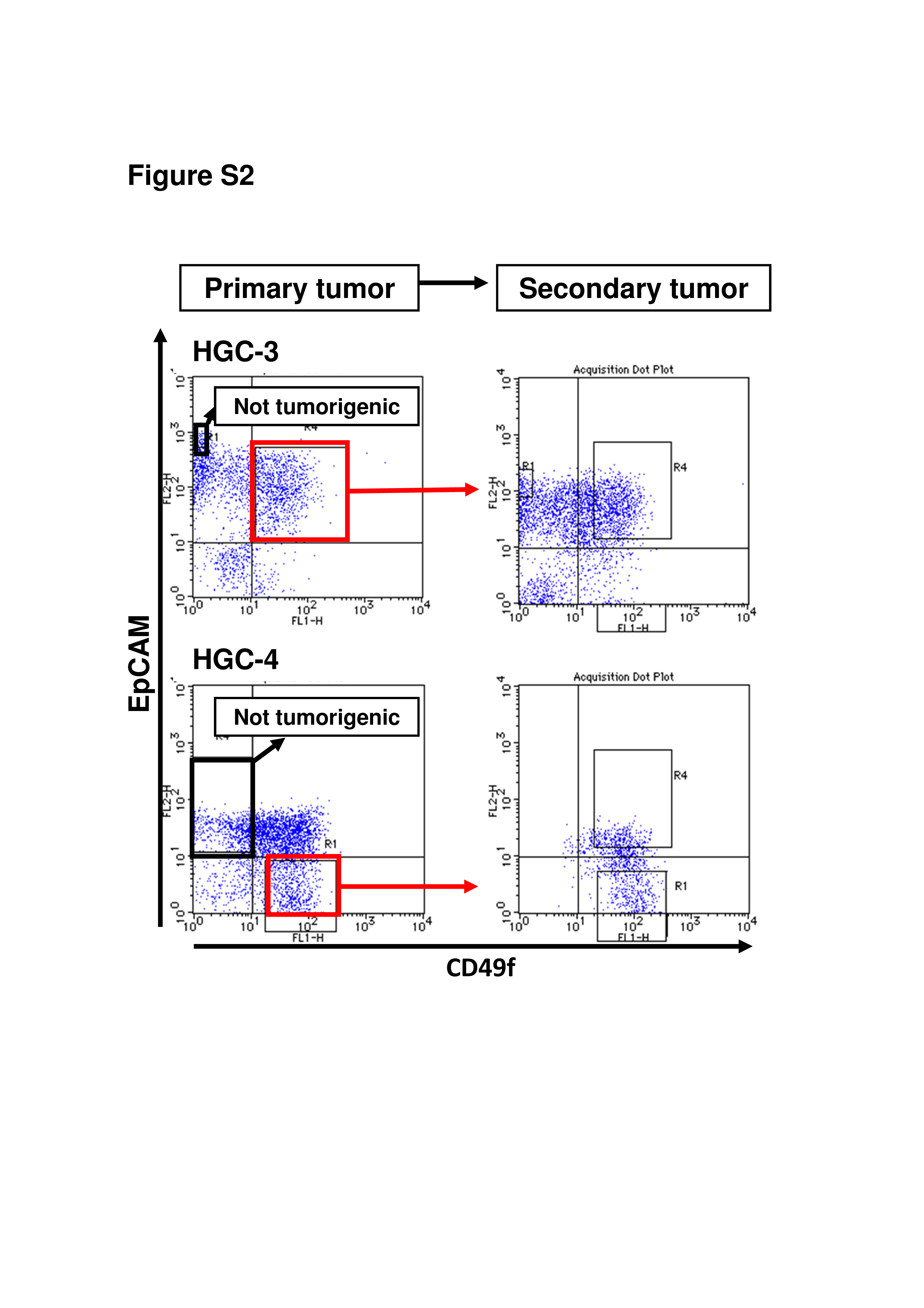

Supplement: Figure S2 — Cell surface antigen profiles are maintained in tumors formed by injection of CD49fhigh cells. Cell surface antigen profiles of HGC-3 and HGC-4 PDTX cells differed greatly, but these profiles of secondary tumors (right panels) formed by injection of CD49fhigh cells (shown by red squares in FACS analyses) into immunodeficient mice were similar to those of primary tumors (left panels). (TIF) [file pone.0072438.s002.tif]

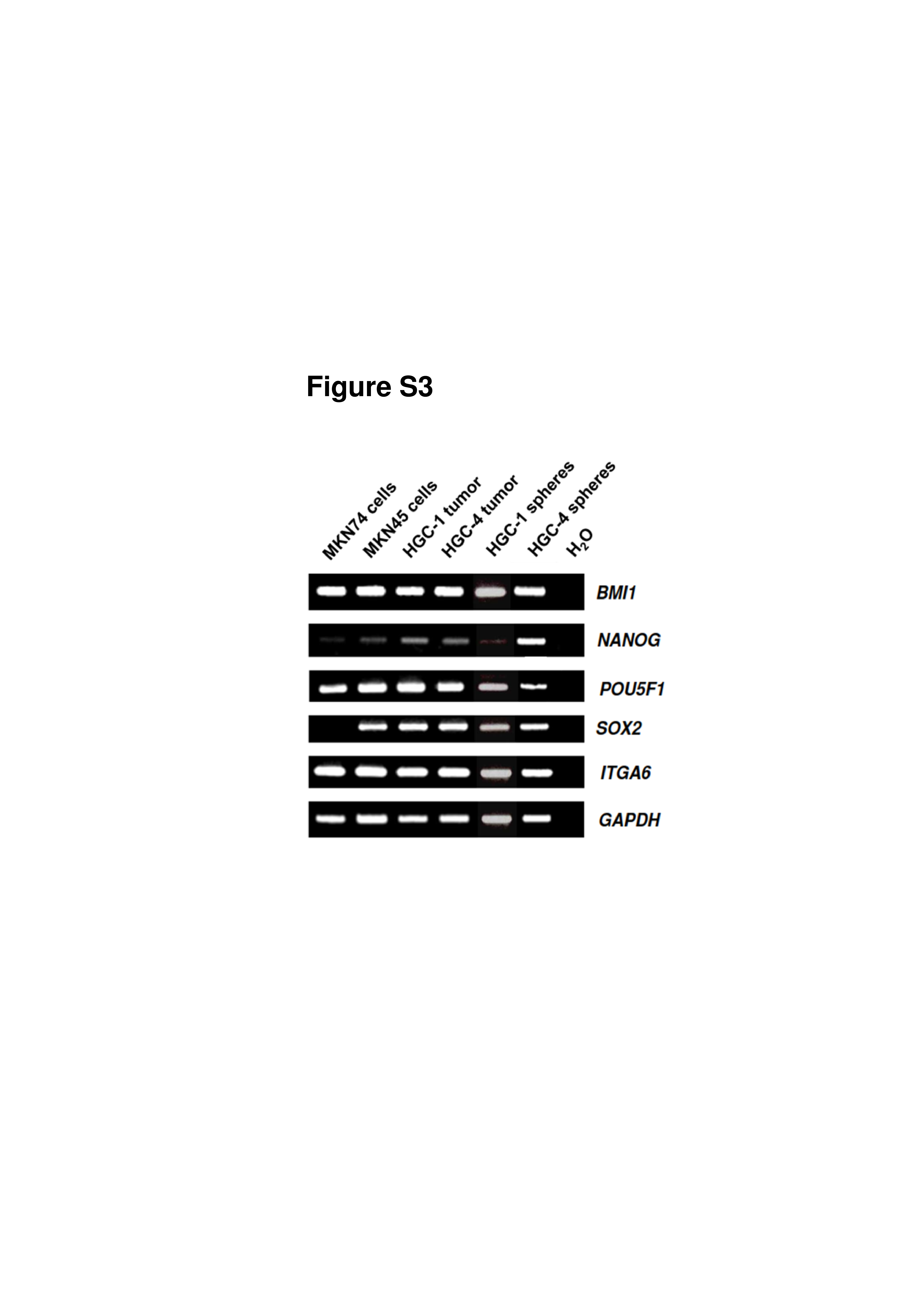

Supplement: Figure S3 — Gene expression profiles of human gastric tumor cell lines, PDTXs and sphere-forming TICs. MKN74 and MKN45 human gastric tumor cell lines, HGC-1 and HGC-4 PDTXs, and HGC-1 and HGC-4 sphere cells formed by culture of unsorted cells expressed stem cell-related genes including BMI1, NANOG, POU5F1, SOX2 and ITGA6 at similar levels though MKN74 cells did not express SOX2, and HGC-4 sphere cells expressed NANOG strongly. (TIF) [file pone.0072438.s003.tif]

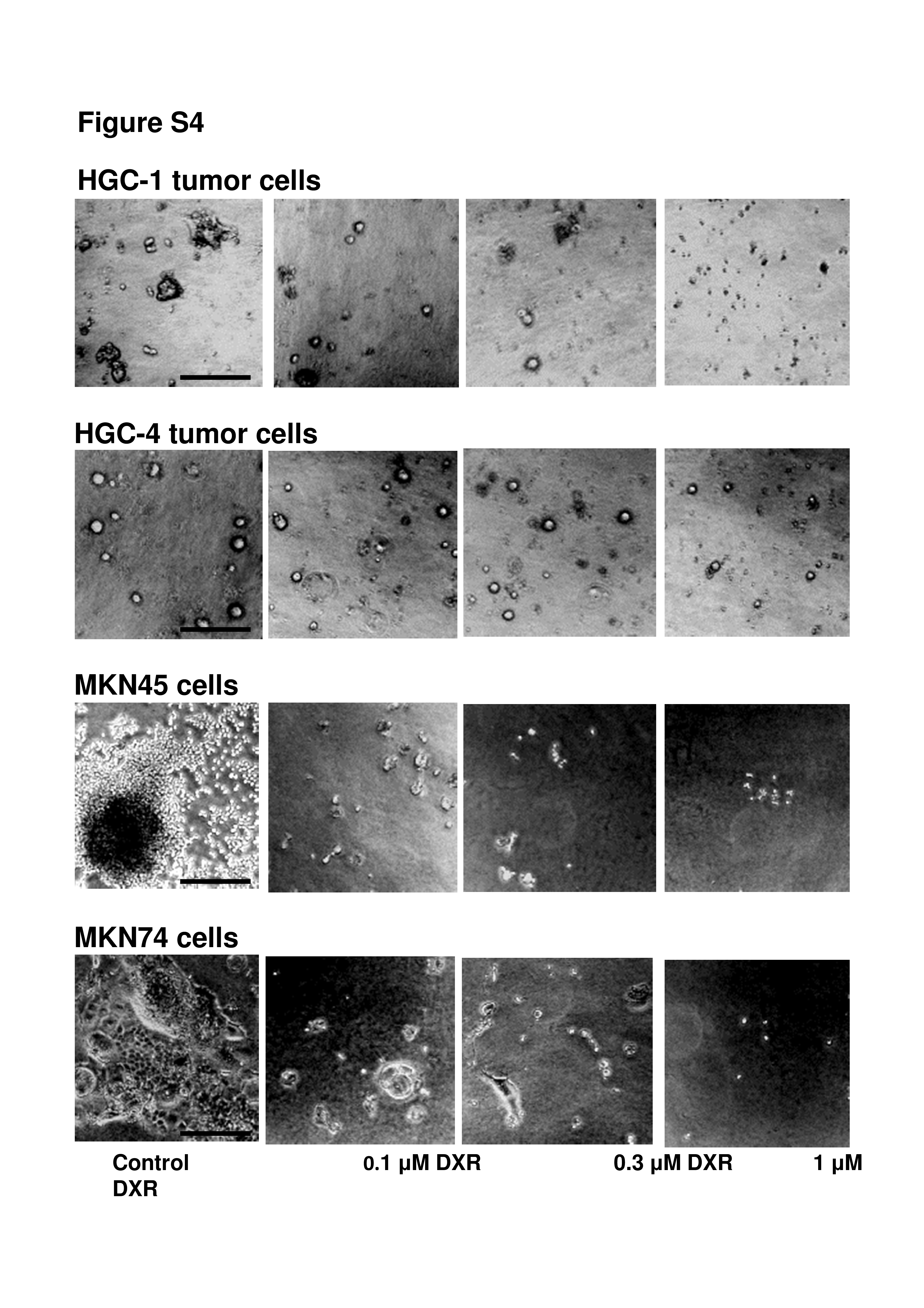

Supplement: Figure S4 — Phase contrast micrographs of doxorubicin (DXR)-treated HGC-1 and HGC-4 tumor cells, MKN45 and MKN74 tumor cell lines on day 14 in vitro. Their growth was quantified by MTT assay and results are shown in Figure 5A. Scale bars represent 200 µm. (TIF) [file pone.0072438.s004.tif]
