# Supplementary material for: CD49fhigh Cells Retain Sphere-Forming and Tumor-Initiating Activities in Human Gastric Tumors
Source: PLoS One. 2013 Aug 28;8(8):e72438. doi: 10.1371/journal.pone.0072438 (PMC3756075; doi:10.1371/journal.pone.0072438)
Supplement: Table S1 — Primer sequences and PCR conditions. (DOCX) [file pone.0072438.s005.docx]

**Table S1. Primer sequences and PCR conditions.**

| Gene | Forward primer | PCR conditions | |
| --- | --- | --- | --- |
|  | Reverse primer | Tm (℃) | cycles |
| *POU5F1* | 5’-TTCTGTAACCGGCGCCAGAAG-3’ | 66 | 35 |
|  | 3’-AATGCATGGGAGCCCAGAG-5’ |  |  |
| *SOX2* | 5’-AACCAAGACGCTCATGAAGAAG-3’ | 60 | 35 |
|  | 3’-CTGCGAGCAGGACATGCTGTAG-5’ |  |  |
| *NANOG* | 5’-GGATCTGCTTATTCAGGACAGC-3’ | 60 | 35 |
|  | 3’-GGTTCAGGATGTTGGAGAGTTC-5’ |  |  |
| *BMI1* | 5’-AGCAGCAATGACTGTGATGCACTTGAG-3’ | 60 | 35 |
|  | 3’-GCTCTCCAGCATTCGTCAGTCCATCCC-5’ |  |  |
| *ITGA6* | 5’-ATGCACGCGGATCGAGTTT-3’ | 60 | 35 |
|  | 3’-TTCCTGCTTCGTATTAACATGCT-5’ |  |  |
| *GAPDH* | 5’- GACCACAGTCCATGCC-3’ | 60 | 25 |
|  | 3’- GTCCACCACCCTGTTG-5’ |  |  |
