# Supplementary material for: CD49fhigh Cells Retain Sphere-Forming and Tumor-Initiating Activities in Human Gastric Tumors
Source: PLoS One. 2013 Aug 28;8(8):e72438. doi: 10.1371/journal.pone.0072438 (PMC3756075; doi:10.1371/journal.pone.0072438)
Supplement: Table S2 — Case description and tumorigenic activity of CD44high and CD44low gastric cancer cells. (DOCX) [file pone.0072438.s006.docx]

**Table S2. Case description and tumorigenic activity of CD44^high^ and CD44^low^ gastric cancer cells**

| Patient number | Age/sex | Site | Bormann  type | Differentiation of tumors | PDTX line | Number of cells injected (number of tumors formed/ number of injection of tumor cells) | | Differentiation of tumors formed by injected cells |
| --- | --- | --- | --- | --- | --- | --- | --- | --- |
|  |  |  |  |  |  | CD44^high^ | CD44^low^ |  |
| 2 | 75/M | Fundic | III | Well | HGC-2 | 3,000 (0/1) | 10,000 (1/1) | Well |
| 3 | 72/M | Pyloric | IV | Poor (Signet ring cell carcinoma) | HGC-3 | 6,000 (1/1) | 6,000 (1/1) | Poor (Signet ring cell carcinoma) |
| 5 | 68/F | Pyloric | II | Poor (Mucinous adenocarcinoma) | HGC-5 | 3,000 (0/1)  6,000 (0/1)  10,000 (1/1)  30,000 (1/3) | 10,000 (3/3)  30,000 (2/2) | Poor (Mucinous adenocarcinoma) |
